# Neuroprotective effect of mitochondrial translocator protein ligand in a mouse model of tauopathy

**DOI:** 10.1186/s12974-021-02122-1

**Published:** 2021-03-19

**Authors:** Lauren H. Fairley, Naruhiko Sahara, Ichio Aoki, Bin Ji, Tetsuya Suhara, Makoto Higuchi, Anna M. Barron

**Affiliations:** 1 Lee Kong Chian School of Medicine, Nanyang Technological University Singapore, Singapore, 308232 Singapore; 2 National Institute of Radiological Science, Chiba City, Chiba Province 263-8555 Japan

**Keywords:** Alzheimer’s disease, Complement, Microglia, Neuroinflammation, Peripheral benzodiazepine receptor, Tau, TSPO, Translocator protein

## Abstract

**Background:**

The translocator protein (TSPO) has been identified as a positron emission tomography (PET)-visible biomarker of inflammation and promising immunotherapeutic target for the treatment of Alzheimer’s disease (AD). While TSPO ligands have been shown to reduce the accumulation of the toxic Alzheimer’s beta-amyloid peptide, their effect on tau pathology has not yet been investigated. To address this, we analyzed the effects of TSPO ligand, Ro5-4864, on the progression of neuropathology in rTg4510 tau transgenic mice (TauTg).

**Methods:**

Brain atrophy, tau accumulation, and neuroinflammation were assessed longitudinally using volumetric magnetic resonance imaging, tau-PET, and TSPO-PET, respectively. In vivo neuroimaging results were confirmed by immunohistochemistry for markers of neuronal survival (NeuN), tauopathy (AT8), and inflammation (TSPO, ionized calcium-binding adaptor molecule 1 or IBA-1, and complement component 1q or C1q) in brain sections from scanned mice.

**Results:**

TSPO ligand treatment attenuated brain atrophy and hippocampal neuronal loss in the absence of any detected effect on tau depositions. Atrophy and neuronal loss were strongly associated with in vivo inflammatory signals measured by TSPO-PET, IBA-1, and levels of C1q, a regulator of the complement cascade. In vitro studies confirmed that the TSPO ligand Ro5-4864 reduces C1q expression in a microglial cell line in response to inflammation, reduction of which has been shown in previous studies to protect synapses and neurons in models of tauopathy.

**Conclusions:**

These findings support a protective role for TSPO ligands in tauopathy, reducing neuroinflammation, neurodegeneration, and brain atrophy.

**Supplementary Information:**

The online version contains supplementary material available at 10.1186/s12974-021-02122-1.

## Background

The mitochondrial translocator protein (TSPO) has been identified as an in vivo biomarker of neuroinflammation detectable via positron emission tomography (PET), and a potential immunotherapy target for the treatment of a range of neurodegenerative diseases including Alzheimer’s disease (AD) [40]. In the AD brain, TSPO expression is upregulated in glia [18, 25], and has been implicated in a wide range of glial functions, including mitochondrial respiration, ATP production, migration, proliferation, phagocytosis, and secretion of cytokines [5, 15, 29]. In mouse models of AD, we and others have shown that TSPO-targeted ligands are protective, reducing the accumulation of the toxic beta amyloid peptide (Aβ), which forms extracellular aggregates in the AD brain [6, 16, 32]. However, to date, no study has investigated the effects of TSPO ligands on the other hallmark pathology of AD, tauopathy, which is most closely associated with the progression of synaptic and neuronal loss and clinical progression of the disease [2, 13].

Tauopathy is comprised of neuronal intracellular aggregates of abnormally phosphorylated tau, a cytoskeletal protein that interacts with microtubules [27]. Tauopathy induces synaptic loss, neuronal dysfunction and death, and although the mechanisms underlying tau-induced neurotoxicity continue to be debated, inflammation is emerging as a key player [35, 38]. Because we have previously demonstrated that TSPO ligands reduce neuroinflammation and reverse Aβ pathology in a mouse model of AD [6], the present study aims to evaluate the effect of the well-characterized TSPO ligand Ro5-4864 (Ro5) on inflammation and neurodegeneration in a mouse model of tauopathy. Here, we use rTg4510 tau transgenic mice (TauTg) expressing a P301L mutant human *tau* gene (P301L) [41]. These TauTg mice exhibit aggressive neuropathology in cortical and hippocampal regions, developing TSPO-positive gliosis, which can be detected by PET and is closely associated with the progression of tau deposition and brain atrophy [8, 26]. We investigate whether treatment with Ro5 attenuates neuroinflammation, tau pathologies, and brain atrophy using clinically translatable, non-invasive neuroimaging approaches including positron emission tomography (PET) and volumetric magnetic resonance imaging (MRI). Inflammation was assessed by PET using the recently described TSPO tracer, 2-[5-(4-^18^F-fluoroethoxy-2-oxo-1,3-benzoxazol-3(2H)-yl)-N-methyl-N-phenylacetamide] (^18^F-FEBMP) [45], while tau aggregates were assessed using the tau-PET tracer, ^11^C-PBB3 [34]. These findings will provide the preclinical foundation for the pursuit of TSPO-targeted therapeutics in AD.

## Methods

### Animals and treatments

All experimentation was approved by the Institutional Animal Care and Use Committee of the National Institutes for Quantum and Radiological Science and Technology, where experimental mice were bred and maintained in Specific Pathogen Free conditions with food and water available ad libitum. Tau transgenic rTg4510 mice (TauTg) expressing mutant human tau (4R0N tau_P301L_) under the tetracycline controlled repressible promoter expressing both the mutant *tau* transgene (4R0N tauP301L) and the tetracycline controlled transactivator were used (Ramsden et al. [37]). NonTg mice were littermates that did not express either transgene. Mice were genotyped by the analysis of DNA isolated from tail clips. At 18-20 weeks of age, TauTg mice were injected (i.p.) with either vehicle (2.5% DMSO, 1% tween in saline; *n* = 3 male and 3 female) or TSPO-ligand Ro5 (3 mg/kg, *n* = 3 male and 4 female) once weekly for a period of 4 months (16 doses). NonTg controls were injected with a vehicle for comparison (*n* = 6 male and 6 female). Since one female vehicle-treated TauTg mouse died in the third week of treatment, and an age-matched female littermate was enrolled in the study as a replacement, no baseline scan data were collected for this mouse. Baseline volumetric MRI, tau-PET, and TSPO-PET were conducted in the first month of the study. In the final month of the study, volumetric MRI, tau-PET, and TSPO-PET was re-evaluated (completion scan), then tissues were collected at ~9 months of age. TSPO-PET was carried out after a 1-week washout period following Ro5 administration. One nonTg mouse was not scanned for MRI at baseline, or ^11^C-PBB3 or ^18^F-FEBMP at the completion time point due to the failure of tail vein catheterization. Data from control groups were used for the evaluation of the performance of a mitochondrial complex-I PET tracer, with scans conducted in mice from all groups at 2 and 7-8 months of age, this data has been reported elsewhere [8]. For tissue collection, mice were anesthetized (50 mg sodium pentobarbital/kg body weight, i.p.) and intracardially perfused with ice-cold, sterile saline. Brains were collected, frozen on dry ice, and stored at −80 °C until analysis.

### In vivo MRI and PET imaging

In vivo MRI and PET scans were carried out on isoflurane-anesthetized mice (3% induction, 1.5-2% maintenance) according to previously described methods [8]. Briefly, transaxial T2-weighted fast spin-echo MR images using a rapid acquisition with relaxation enhancement sequence were acquired using a 7.0-T horizontal MRI scanner (Magnet: 40 cm bore, Kobelco & JASTEC, Japan; Console: Avance-I, Bruker Biospin, Germany) with a volume coil for transmission (Bruker BioSpin) and a quadrature surface coil for reception (Rapid Biomedical). Manual segmentation to define anatomical regions of interest (cortex, hippocampus, and cerebellum) was performed with reference to the mouse brain atlas of Paxinos and Franklin using the PMOD software (version 3.4; PMOD Technologies, Inc., Zurich, Switzerland).

For PET studies, ^11^C-PBB3 and ^18^F-FEBMP were prepared according to previously published methods [34, 45, 47]. The molar activity of the end-product was 61.1 ± 7.5 GBq/μmol and 290.5 ± 1.0 GBq/μmol for ^11^C-PBB3 and ^18^F-FEBMP, respectively. Anesthetized mice were administered with either ^11^C-PBB3 (30.5 ± 0.85 MBq) or ^18^F-FEBMP (34.2 ± 0.7 MBq) via tail vein catheter while in a microPET Focus 220 animal scanner (Siemens Medical Solutions). A 90 min (^18^F-FEBMP) or 60 min (^11^C-PBB3) 3D list mode emission scan was started simultaneously with radioligand injection. List-mode data were sorted into 3D sinograms then Fourier rebinned into 2D sonograms with time frame reconstruction of 1 min × 4 frames, 2 min × 8 frames, and 5 min × 14 frames. Summation images were reconstructed with maximum a posteriori reconstruction, and dynamic images were reconstructed with filtered back-projection using a 0.5-mm Hanning filter. Dynamic PET images were merged to the individual MRI data by an experimenter blinded to treatment conditions, and tracer uptake in each VOI was estimated as the percent of injected dose per mL (%ID/mL). Because the cerebellum is unaffected by pathology, the cerebellum was used as a reference for calculation of the target-to-reference ratio [8, 9].

### Immunohistochemistry

Fresh frozen hemibrains were sectioned in the sagittal plane at 20 μm by a cryostat. Sections were fixed in 4% paraformaldehyde and labeled using antibodies directed against ionized calcium-binding adaptor molecule-1 (IBA-1, rabbit polyclonal, WAKO), NeuN (Abcam ab177487), C1q (Abcam ab182451), AT8 (mouse monoclonal, Endogen), and TSPO (rabbit monoclonal, Abcam). All staining, imaging, and analysis parameters were maintained consistently between samples for each marker. Images were captured using a fluorescence microscope/digital camera (BZ-X700, Keyence) and analyses were performed in an experimenter-blinded manner using ImageJ. Images were background subtracted using rolling ball radius of 50 pixels prior to analysis or use in figures. For measurement of CA1 width, images of NeuN staining in the CA1 region were captured at ×20 magnification and three lines per image were drawn across the CA1 region at equidistant intervals using the straight line tool, and the average length in pixels was measured, as previously described [17]. NeuN staining and CA1 width analysis was repeated twice on non-consecutive sections for each animal and an average used for analysis of group-wise differences. For quantification of IBA-1 and TSPO, an image of the frontal cortex and hippocampus was captured in two sections per animal and after background subtraction, 8-bit images were thresholded at 25-255 for IBA-1 or 20-255 for TSPO and percent area measured. For quantification of AT8 and C1q, the entire section was imaged using the automated image stitching and regions of interest were manually drawn around the hippocampus and cortex using anatomical landmarks. Images were thresholded to create a binary image identifying positive and negative immunolabeling, and the percent area of positive labeling in the regions of interest measured, as previously described [9]. Brightness is linearly adjusted in images presented in figures, with adjustment applied equally to all samples.

### Western blot

Western blotting was performed as previously described (Sahara et al. [42]). Briefly, cortical and hippocampal regions were combined and homogenized in Tris-buffered saline (TBS, 50 mM Tris/hydrochloric acid, pH 7.4, 274 mM sodium chloride, 5 mM potassium chloride; ×10 vol/tissue wet weight) containing 1% protease inhibitor mixture (Sigma, St. Louis, MO), 1% phosphatase inhibitor cocktail I and II (Sigma), and 1 mM phenylmethylsulfonyl fluoride. Homogenates were centrifuged at 27,000×*g* for 20 min at 4 °C, and the supernatant was diluted in sodium dodecyl sulfate-sample buffer containing β-mercaptoethanol (2.5%) and heat-treated at 55 °C for 15 min. Samples were separated by gel electrophoresis in 10% Tris-glycine sodium dodecyl sulfate-polyacrylamide gels (Nacalai Tesque) and transferred to nitrocellulose membranes (BioRad Laboratories, Hercules, CA). Membranes were incubated with a blocking solution containing 5% skim milk and 0.1% Triton X-100 in TBS for 30 min. Membranes were then probed with anti-Tau12 (Invitrogen, Cat #MAB2241) and glyceraldehyde-3-phosphate-dehydrogenase (GAPDH, Sigma-Aldrich, Cat # MAB374) antibodies overnight at 4 °C, followed by anti-mouse Immunoglobulin G (1:5000; Jackson ImmunoResearch) for 1 h at RT. Chemiluminescent signals were detected using an enhanced chemiluminescence system (ECL PLUS kit; PerkinElmer), and immunoreactivity was visualized by a computer-linked LAS-4000 BioImaging Analyzer System (Fujifilm, Tokyo, Japan). Band intensities were quantified using the NIH ImageJ software. To compare tau levels across different mouse brains, the relative amount of tau protein was normalized against GAPDH levels. All blots were repeated twice, and the average of two blots was calculated.

### Cell culture and flow cytometry

The immortalized murine microglial BV-2 cell line was grown and maintained in Dulbecco’s modified Eagle serum (DMEM) containing 10% fetal bovine serum and 1% penicillin-streptomycin at 37 °C in 5% CO_2._ BV-2 cells were collected, and 1 × 10^5^ cells were plated in 6-well plates overnight. The medium was removed, and cells were stimulated with either vehicle (1% ethanol) or 10nM Ro5 (Sigma Aldrich, C5174) diluted in DMEM-Ham’s F12 containing 1% penicillin-streptomycin for 24 h, followed by stimulation with 100ng/ml LPS or vehicle for 2h. Cells were then trypsinized and centrifuged, and the resulting pellet was fixed in 4% paraformaldehyde for 10 min, followed by permeabilization with 0.1% Triton X-100 in phosphate-buffered saline for 5 min. C1q was labeled using an anti-C1q antibody (Abcam, ab182451), and fluorochrome-conjugated anti-rabbit secondary antibody (Invitrogen, A11034), and fluorescence was detected by flow cytometry. Mean fluorescent intensity was analyzed using the FlowJo software (Tree Star, Ashland, OR).

### Data analysis

Data were analyzed using the Statistical Package for Social Sciences (SPSS: version 25; SPSS Inc., IL, USA), Primer version 6 (PRIMER-E, Auckland, New Zealand), and the R environment (R Core Team. R [39]) with the lme4 package (Bates, Maechler, Bolker, and Walker  [10]) and plots were generated using GraphPad Prism 8.2.1 (GraphPad Software).

Homoscedasticity and normality was assessed using diagnostic residual plots, Levine’s, and Shapiro-Wilk tests. Parametric data were analyzed by ANOVA. MRI and PET data was analyzed using a 3×2×3 mixed-model ANOVA including brain region (CTX, HIP, CB) and treatment time point (baseline = 5 months; completion = 9 months) as within-subject factors and group (nonTg, TauTg+ Veh, TauTg + Ro5) as the between subjects factor. AT8 was analyzed by a 2×3 mixed-model ANOVA, including brain region (CTX and HIP) as the within-subjects factors and treatment group (nonTg, TauTg + Veh, TauTg + Ro5) as the between subjects factor. Hippocampal width measured by NeuN was analyzed by one-way ANOVA. Significant factors or interactions were further explored using least squares difference post-hoc comparisons. Associations between variables were evaluated using Spearman’s rank correlation coefficient. The effect of treatment on inflammatory markers TSPO, IBA-1, and C1q measured by immunoreactivity was analyzed by permutational multivariate analysis of variance (PERMANOVA), including treatment group and region as fixed factors. Between sample similarity was computed based on Euclidean distances, and analysis was based on 9999 unrestricted permutations of raw data using type III (partial) sum of squares, and the resulting *F* statistic is quoted as “pseudo-F” and *p* statistic “*p*_perm_” [1]. PERMANOVA analysis of inflammatory markers was followed by distance-based redundancy analysis (dbRDA) to explain the observed differences in the inflammatory response of the treatment groups. C1q flow cytometry data was analyzed by one-way ANOVA.

## Results

### TSPO ligand Ro5-4864 attenuates brain atrophy and neuronal loss in TauTg mice

**Fig. 1:**
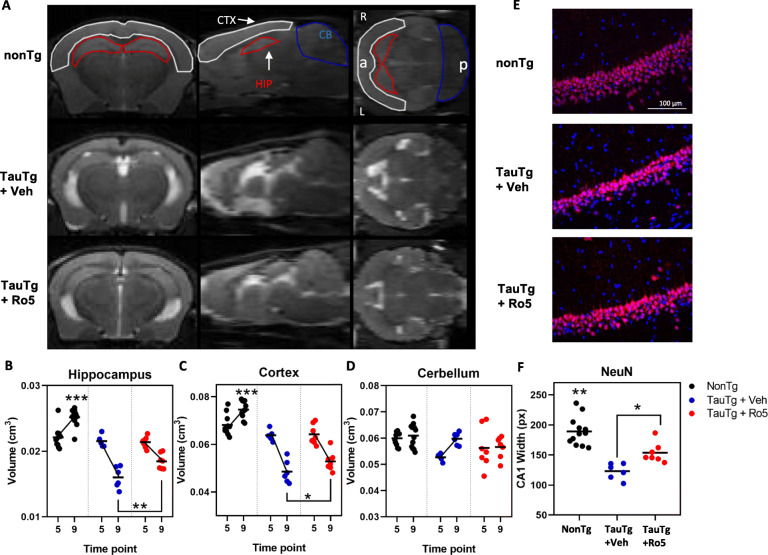
TSPO ligand, Ro5-4864, attenuates brain atrophy and neuronal loss in TauTg mice. **a** Representative T2-weighted RARE coronal (left), sagittal (middle), and horizontal (right) images of 9-month-old nonTg and TauTg mouse brains. Brain regions of cerebral cortex (CTX), hippocampus (HIP), and cerebellum (CB) are labeled in the sagittal slice of nonTg mice. Orientations of slice are indicated as “a” (anterior), “p” (posterior), R (right), and L (left). **b**-**d** Hippocampal (**b**), cerebral cortical (**c**), and cerebellar (**d**) volumes at baseline (5 m) and completion (9 m) in nonTg (*N* = 12) and TauTg mice treated with either vehicle (*N* = 6) or Ro5 (*N* = 7). **e** Representative images of NeuN (red) and DAPI (blue) immunoreactivity in the CA1 region of 9-month old nonTg and TauTg mice treated with either vehicle or Ro5. **f** Quantification of NeuN-labeled CA1 width in the hippocampus of nonTg (*N* = 12) and TauTg mice treated with either vehicle (*N* = 6) or Ro5 (*N* = 7). Mixed ANOVA was used except in (**f**), where one-way ANOVA was used. Individual data points plotted for each group and the group mean indicated (black bar). ***p < 0.001, **p < 0.01, *p < 0.05

To evaluate the effect of TSPO ligand Ro5 treatment on brain atrophy, cortical, hippocampal, and cerebellar volumes were measured longitudinally in vivo by volumetric MRI at baseline (5 months) and following treatment (9 months; Fig. 1a). Mixed ANOVA revealed an interaction between group, treatment time point, and brain region on brain volume (*F*_(2, 20)_ = 12.322 , *p* < 0.001; Fig. 1a-d). At baseline, no significant differences in brain volume were observed between groups (hippocampus: nonTg vs TauTg + veh *p =* 0.426; cortex: nonTg vs TauTg + veh *p =* 0.57); however, by 9 months of age TauTg mice showed reduced hippocampal (*p* < 0.001, Fig. 1b) and cortical (*p* < 0.001, Fig. 1c), but not cerebellar volumes (*p =* 0.523; Fig. 1d) compared to nonTg mice. While nonTg mice exhibited a modest 10-15% increase in hippocampal and cortical volumes between 5 and 9 months, vehicle-treated TauTg mice exhibited a loss of more than 25% of hippocampal and 20% of cortical volume over this same duration. Treatment with Ro5 markedly attenuated atrophy between 5 and 9 months, halving both hippocampal and cortical volumetric loss compared to vehicle-treated TauTg mice (hippocampus: *p* = 0.005; cortex: *p* = 0.021, Fig. 1b, c).

To corroborate in vivo volumetric MRI findings, neuronal viability was assessed in sections from scanned mice at 9 months of age by immunohistochemistry for the neuronal marker, NeuN (Fig. 3e, f). A significant main effect of group was observed (one way ANOVA; *F*_(2,22)_ = 23.393 *p <* 0.001). Consistent with volumetric MRI data, hippocampal CA1 pyramidal layer thickness was reduced in TauTg compared to nonTg mice (CA1 width: nonTg vs TauTg + veh *p <* 0.001; nonTg vs TauTg + Ro5 *p* = 0.003). However, a neuroprotective effect of Ro5 treatment was observed, with a modest increase in CA1 width in TauTg mice treated with Ro5 compared to vehicle (*p* = 0.028, Fig. 1f).

### No benefit of TSPO ligand Ro5-4864 on tau deposition in TauTg mice

**Fig. 2:**
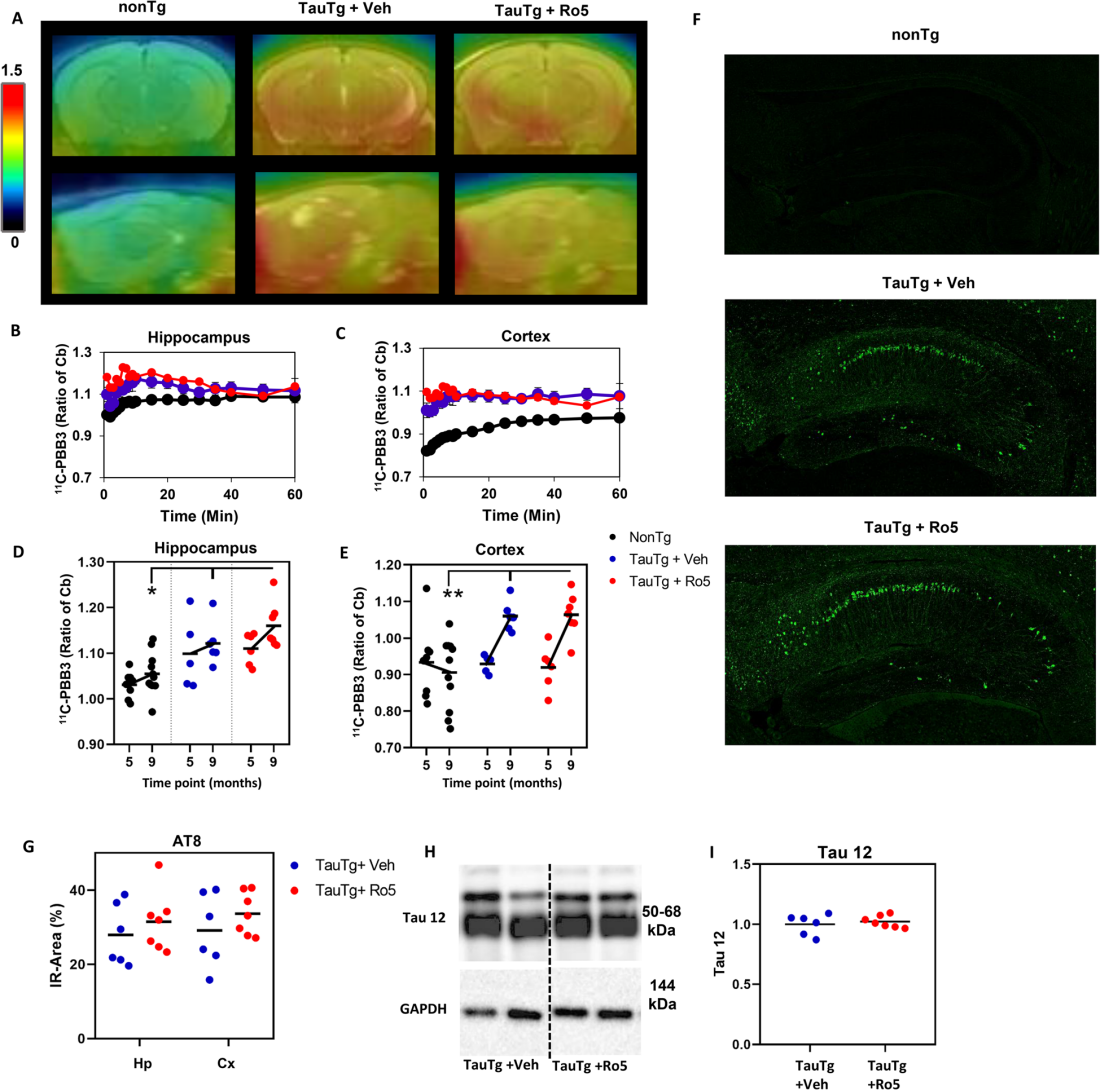
No benefit of TSPO ligand on tau depositions measured by in vivo ^11^C-PBB3-PET. **a** Representative orthogonal views of ^11^C-PBB3 uptake distribution in brains of nonTg and TauTg mice following treatment with either vehicle or Ro5. Images were generated by averaging dynamic scan data across 60 min after intravenous injection of ^11^C-PBB3 and were overlaid on individual MRI data. **b**, **c** Time activity curves of the ratio of cortical (**b**) and hippocampal (**c**) ^11^C-PBB3 mean ± SEM uptake relative to cerebellum (cb) in nonTg (*N* = 11) and TauTg mice following treatment with either vehicle (*N* = 6) or Ro5 (*N* = 7). **d**, **e** Average ratio ^11^C-PBB3 uptake (relative cerebellum, cb) in cortex (**d**) and hippocampus (**e**) of nonTg and TauTg mice at baseline (5 m) and following treatment with either vehicle or Ro5-4864 (9 m). Individual data points plotted for each group and the group mean indicated (black bar). **f** Quantification of AT8 immunoreactivity (IR) in the hippocampus (Hp) and cortex (cx) of TauTg mice treated with either Ro5 (*N* = 7) or vehicle (*N* = 6). Individual data points plotted for each group and the group mean indicated (black bar). **g** Representative images of AT8 immunoreactivity in the hippocampus of nonTg and TauTg mice treated with either vehicle or Ro5. **h** Representative western blots showing total tau (tau-12) and GAPDH in brains from TauTg mice treated with either vehicle or Ro5. **i** Quantification of total tau corrected against GAPDH in TauTg mice treated with either vehicle (*N* = 6) or Ro5 (*N* = 7). Mixed ANOVA was used except in (**i**), where unpaired parametric *T* test was used. **p < 0.01, *p < 0.05

To determine if Ro5 treatment attenuated neuronal loss through effects on tau pathogenesis, tau accumulation was measured longitudinally in vivo by ^11^C-PBB3-PET at baseline (5 m) and following treatment (9 m, Fig. 2a). Mixed ANOVA revealed a significant interaction between group, treatment time point, and region on ^11^C-PBB3 uptake (*F*_(2,17)_ = 5.795, *p* = 0.012; Fig. 2b). At baseline, increased ^11^C-PBB3 uptake was observed in TauTg mice compared to nonTg mice in the hippocampus (TauTg + veh *p* = 0.017; TauTg + Ro5 *p* = 0.005), but not cortex (TauTg + veh: *p =* 0.913; TauTg + Ro5 *p =* 0.885). At 9 months of age, increased ^11^C-PBB3 uptake was observed in TauTg mice treated with both vehicle and Ro5 in the hippocampus (nonTg v TauTg + Veh *p* = 0.017; nonTg v TauTg + Ro5 *p* = 0.001) and cortex (nonTg v TauTg + Veh *p* = 0.002; nonTg v TauTg + Ro5 *p* < 0.001; Fig. 2a-e). No differences between vehicle- and Ro5-treated TauTg mice were observed (hippocampus: *p* = 0.239; cortex: *p =* 0.566; Fig. 2d, e).

In vivo tau PET findings were confirmed in sections from scanned mice by immunohistochemistry using the phosphorylated tau antibody, AT8 (Fig. 2f, g). No differences in AT8 immunoreactivity between regions was observed (mixed ANOVA; *F*_(1,10)_ = 1.593, *p* = 0.235) and, consistent with ^11^C-PBB3 signals, no difference in AT8 immunoreactivity was observed between vehicle and Ro5-treated TauTg mice (*F*_(1,10)_ = 0.794, *p* = 0.394; Fig. 2f, g). No AT8 immunoreactivity was observed in nonTg mice. To determine if Ro5 had any effect on expressions of tau proteins, the total tau was assessed in TBS-extractable fractions from combined cortical and hippocampal homogenate by western blot using antibodies directed against N-terminus of human tau (Tau 12). No difference in total tau levels were observed between vehicle- and Ro5-treated TauTg mice (*t*_(11)_ = −0.542, *p* = 0.226; Fig. 2h, i).

### TSPO ligand attenuates neuroinflammation in TauTg mice

**Fig. 3:**
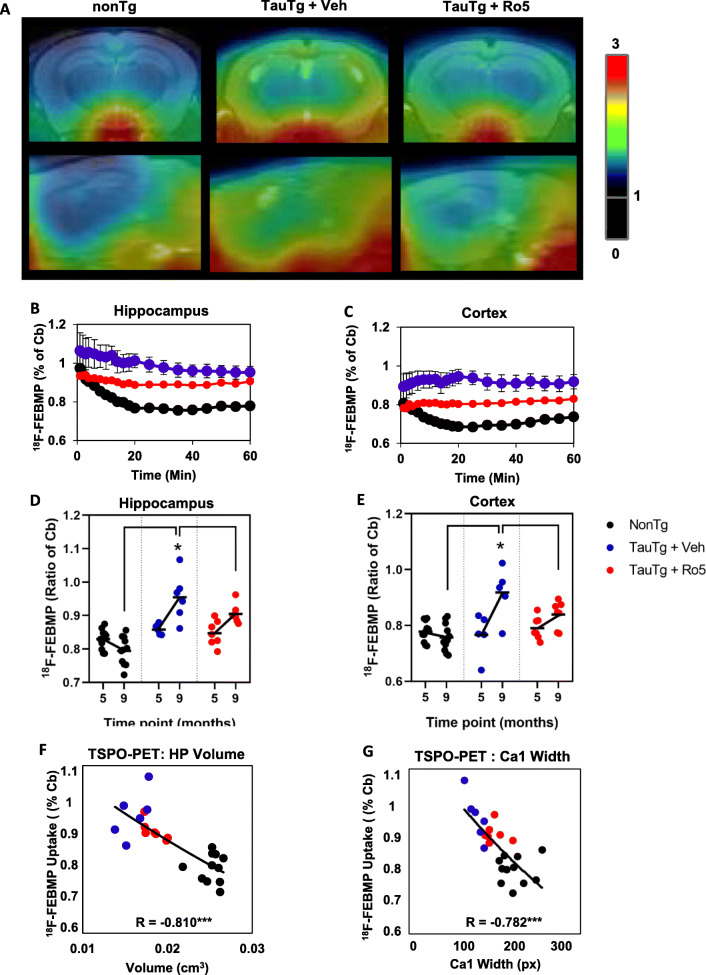
Inflammatory signals detected by in vivo PET with ^18^F-FEBMP in TSPO ligand treated TauTg mice. **a** Representative orthogonal views of ^18^F-FEBMP uptake distribution in brains of nonTg and TauTg mice following treatment with either vehicle or Ro5. Images were generated by averaging dynamic scan data across 90 min after intravenous injection of ^18^F-FEBMP and were overlaid on individual MRI data. **b**, **c** Time activity curves of the ratio of mean ± SEM hippocampal (**b**) and cortical (**c**) ^18^F-FEBMP uptake relative to cerebellum (cb) in nonTg (*N* = 11) and TauTg mice following treatment with either vehicle (*N* = 6) or Ro5 (*N* = 7). **d**, **e** Average ratio ^18^F-FEBMP uptake (relative cerebellum, cb) in hippocampus (**d**) and cortex (**e**) of nonTg and TauTg mice at baseline (5 months) and following treatment with either vehicle or Ro5-4864 (9 months). Individual data points plotted for each group and the group mean indicated (black bar). **f**, **g** Association between hippocampal TSPO-PET signals and measures of neurodegeneration including hippocampal MRI volume (**f**) and CA1 width (**g**) assessed at study completion. Mixed ANOVA was used except in (**f**, **g**), where Spearman’s correlation was used *p < 0.05. ***p < 0.0001

The effect of TSPO ligand treatment on in vivo TSPO signals were measured longitudinally by PET using ^18^F-FEBMP, comparing baseline (5 months) and post-treatment time points (9 months, Fig. 3). An interaction between group and treatment time point was observed on ^18^F-FEBMP signals in the hippocampus and cortex (Mixed ANOVA, *F*_(2,20)_ = 12, *p* < 0.001). At baseline, no differences in ^18^F-FEBMP signals were observed between nonTg and TauTg mice (nonTg vs TauTg + veh: *p =* 0.281; nonTg vs TauTg + Ro5: *p* = 0.2). At 9 months of age, ^18^F-FEMBP signals were significantly increased in vehicle treated TauTg mice compared to both Ro5 treated TauTg mice (*p* = 0.029) and nonTg mice (*p* < 0.001, Fig. 3d, e). Hippocampal TSPO-PET signals at study completion were strongly associated with measures of neurodegeneration including atrophy assessed by MRI (*r* = −0.810, *p* < 0.0001; Fig. 3f) and CA1 width (*r* = −0.782, *p* < 0.0001; Fig. 3g).

**Fig. 4:**
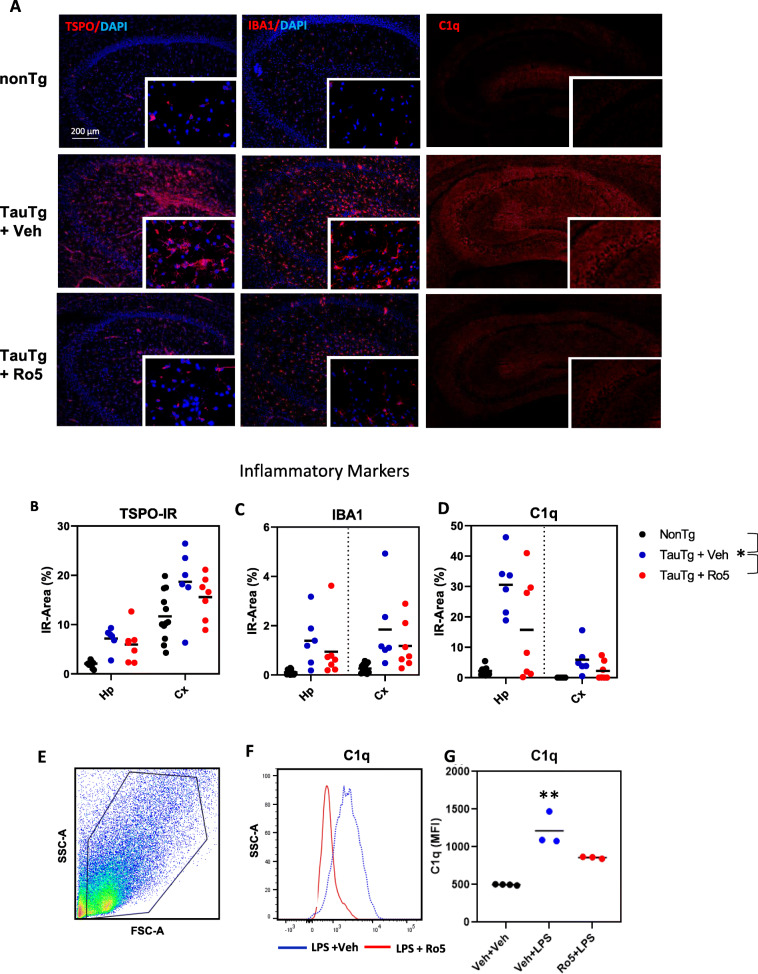
TSPO ligand reduces hippocampal neuroinflammation in TauTg mice. **a** Representative images of TSPO (*left panel*), IBA-1 (*middle panel*), and C1q (*right panel)* immunoreactivity in the hippocampus of 9-month-old nonTg, and TauTg mice treated with either vehicle or Ro5. Inset shows digitally magnified view of image. **b**-**d** Quantification of immunoreactivity of the inflammatory markers, TSPO (**b**), IBA-1 (**c**), and C1q (**d**) in the hippocampus and cerebral cortices of nonTg (*N* = 12) and TauTg mice treated with either Ro5 (*N* = 7) or vehicle (*N* = 6). Individual data points plotted for each group and the group mean indicated (black bar). PERMANOVA, *p_perm_ < 0.03. **e** Representative gating (*left*) and flow cytometry histogram of C1q levels (*right*) in unstimulated BV2 cells (black) or following stimulation with LPS after pretreatment with either vehicle (blue) or Ro5 (red). MFI, mean fluorescent intensity. SSC, side scatter. FSC, forward scatter. **f** Quantification of C1q binding in BV2 cells (*n*=3-4/group/experiment, single experiment shown, experiment replicated 3 times). One-way ANOVA. **p = 0.003

In vivo neuroinflammatory signals measured by TSPO-PET were corroborated in sections from scanned mice by immunohistochemistry for inflammatory markers including TSPO, the microglial marker IBA-1, and regulator of the complement cascade, complement component C1q (Fig. 4a). Inflammatory marker immunoreactivity was correlated with in vivo TSPO-PET signals in the hippocampus (TSPO: *r* = 0.726, *p* < 0.0001; IBA-1: hippocampus *r* = 0.754, *p* < 0.0001; C1q: *r* = 0.572, *p* = 0.003; Suppl. Fig. 1A-C). Multivariate analysis of TSPO, IBA-1, and C1q immunoreactivity revealed significant differences in inflammatory markers across groups (PERMANOVA, pseudo-F_(2, 49)_ = 19.24, *p*_perm_ = 0.0001), with vehicle-treated TauTg mice significantly different compared to both nonTg (*p*_perm_ = 0.0001) and Ro5-treated TauTg groups (*p*_perm_ = 0.03). Mean levels of TSPO, IBA-1, and C1q were increased in TauTg compared to nonTg mice, with levels of IBA-1 and C1q increasing over tenfold in vehicle treated TauTg compared to nonTg mice. Treatment of TauTg mice with Ro5 reduced mean levels of the three inflammatory markers measured, although a large amount of individual variability within groups was noted (Fig. 4b-d). Distance-based redundancy analysis (dbRDA) indicated that most (75%) of the variation between groups in inflammatory markers was closely correlated with C1q (0.998). Measures of neurodegeneration in both the hippocampus and cortex were negatively associated with inflammatory markers, particularly IBA-1 and TSPO (Table 1).

**Table 1:** Markers of neurodegeneration strongly associated with inflammatory markers. Spearman’s correlation between inflammatory markers, IBA-1, TSPO, and C1q, measured by immunoreactivity and measures of neurodegeneration assessed by MRI volume and NeuN immunoreactivity in hippocampus (Hp) and cortex (Cx)

Region	Inflammatory marker	Neurodegeneration marker	Correlation	
			***r***	***p***
Hp	IBA-1	MRI volume	-0.792	<0.0001
	TSPO		-0.833	<0.0001
	C1q		-0.538	0.005
	IBA-1	CA1 Width	-0.679	<0.0001
	TSPO		-0.772	<0.0001
	C1q		-0.588,	0.002
Cx	IBA-1	MRI volume	-0.702	<0.0001
	TSPO		-0.538	0.006
	C1q		-0.795	<0.0001

Since C1q is almost exclusively produced by microglia in the brain [20, 21], we examined the effect of Ro5 on C1q levels in the immortalized microglial cell line, BV2, to determine if Ro5 attenuated C1q levels in the TauTg mice by affecting the microglial inflammatory response. BV2 microglial cells were pretreated with either Ro5-4864 or vehicle, then stimulated with LPS to induce inflammation. LPS markedly increased C1q levels, an effect that was inhibited by Ro5 pre-treatment (ANOVA, *F*_(2,7)_ = 24.825, *p* < 0.001; Veh vs Veh + LPS: *p* < 0.001; Veh + LPS vs Ro5 + LPS: *p* = 0.028; *n* = 3-4/group in each experiment, experiment repeated 3 times; Fig. 4e-g).

## Discussion

Here, we demonstrate that the TSPO ligand Ro5 is protective in a mouse model of tauopathy, reducing markers of neurodegeneration, which was strongly associated with inflammatory signals measured in vivo and in end-point tissues. Further, we demonstrate the potential usefulness of the recently described TSPO-PET tracer, ^18^F-FEBMP, for evaluating efficacy of novel immunotherapeutics for AD. Taken together, these findings indicate that TSPO is a promising target for the development of biomarker and immunomodulatory strategies in AD.

In the current study, we observed a marked effect of Ro5 treatment on neurodegeneration in a model of tauopathy, with atrophy and neuronal loss reduced by half. The magnitude of this neuroprotective effect is similar to that observed through complete repression of the tau transgene using doxycycline treatment from 4 months of age [12]. Although the mechanism of neuroprotection in the current study is not clear, we propose that the neuroprotective effect was downstream of tau accumulation since we did not observe any significant benefit on measures of tauopathy. However, we cannot rule out that the neuroprotective effect of TSPO ligand treatment may have been mediated through non-tau-related neuronal death mechanisms since recent studies have demonstrated that neuronal loss in this TauTg model is not solely the result of mutant tau overexpression, but disruption and partial deletion of a number of other genes at the insertional site of the tau transgene also contributes [22].

Since TSPO is highly expressed in microglia and has been identified as an immunomodulator, we hypothesized that Ro5 may have mediated neuroprotection via action on microglia. Previous studies have demonstrated TSPO ligands inhibit detrimental inflammatory profiles in microglia, including in response to LPS [14, 46], while in vivo neuroprotective effects linked to immune modulation by TSPO ligands have been described in other models of injury including a model of Parkinson’s disease [23] and retinal degeneration [43]. Consistent with this hypothesis, here we found that markers of neuroinflammation were strongly associated with degree of atrophy and neuronal loss. Further, we demonstrated in vitro that the TSPO ligand Ro5 acts on immortalized microglial cell lines to reduce C1q expression in response to inflammation. C1q is the recognition component that initiates the classical complement cascade system, identified as a marker of detrimental inflammatory phenotypes associated with LPS-induced inflammatory programs in microglia and is upregulated in TauTg mice [19, 34]. Future studies could further investigate interactions between TSPO, C1q, and tau-induced neuronal death.

Although TSPO is highly expressed in activated microglia [28, 33], recent studies have also demonstrated that neurons express TSPO, albeit at low levels, and may too be a direct target for TSPO therapeutics [7, 36]. Further, recent studies have demonstrated that TSPO ligands can protect cultured neurons against tau and Aβ induced toxicity by promoting mitochondrial fitness [24, 31]. Future studies could use conditional TSPO knockout models to address the relative neuronal versus microglial cellular targets of TSPO therapeutic effects in models of neurodegeneration, which may provide more insight into mechanism of neuroprotection.

Interestingly, despite the protective effect of Ro5 against atrophy and neurodegeneration, no benefit was observed on tau depositions. It is possible the beneficial effect of Ro5 might be underestimated due to an improved survival of tangle-bearing neurons following the treatment. Further, other studies suggest that only the most robust inhibition of microglia is effective in modulating tau pathology. For instance, previous studies in TauTg mice have found that while near-complete depletion of microglia successfully reduced tauopathy [3], partial depletion of microglia had no effect on tauopathy [11]. Therefore, higher or more frequent dosing may be required to alter tauopathy with TSPO ligands. Additionally, immunomodulatory strategies may be more effective when initiated early in the disease process. For example, microglial depletion protected against neurodegeneration and tau accumulation when treatment was started prior to the onset of neuronal degeneration [44]; however, other studies that initiated treatment after neuronal loss had begun reported no protective effects [11]. While our current study provides proof-in-principle that TSPO-targeted drugs are protective against tau-induced neurodegeneration, future studies could address optimal dosing and early versus late intervention strategies.

Of note, other studies targeting microglial function have also reported beneficial neuroprotective effects in the absence of altered tauopathy. For example, tyrosine motif binding protein (TYROBP) knockout resulted in reduced C1q, improved synaptic function and learning and memory outcomes, despite worsening of tauopathy [4]. TYROBP has been identified as a key regulator of the complement family in late-onset AD [48] and is implicated in the differentiation of the protective disease-associated microglia (DAM) phenotype [30]. These findings suggest microglial functions affecting tau accumulation versus synaptic loss are not necessarily overlapping, and importantly, highlights that although damaged by tauopathy, synapses and neurons remain functional if they are spared from neuronal death.

## Conclusions

Here, we report the first evidence to date that TSPO ligands exert protective effects in a mouse model of tauopathy, attenuating brain atrophy and reducing neuroinflammation. While many new generation, safe TSPO ligands have been developed for in vivo PET imaging in humans, our findings suggest these ligands may also have therapeutic potential for the treatment of AD.

## Supplementary Information

**Additional file 1: Supplementary Figure 1.** Association between *in vivo* hippocampal TSPO signals measured using ^18^F-FEBMP-PET and inflammatory marker immunoreactivity. ^18^F-FEBMP-PET signals and immunoreactivity (IR) of TSPO (A), IBA-1 (B) and C1q (C).

## Data Availability

The dataset(s) supporting the conclusions of this article are available in the Nanyang Technological University Institutional repository https://dr.ntu.edu.sg/.

## Abbreviations

Aβ: Beta amyloid
AD: Alzheimer’s disease
CA1: Cornu Ammonis-1
C1q: Complement component 1q
^11^C-PBB3: 2-((1E,3E)-4-(6-((11)C-methylamino)pyridin-3-yl)buta-1,3-dienyl) benzo[d]thiazol-6-ol
DAM: Disease-associated microglia
dbRDA: Distance-based redundancy analysis
DMEM: Dulbecco’s modified Eagle serum
DMSO: Dimethyl sulfoxide
^18^F-FEBMP: 2-[5-(4-^18^F-fluoroethoxy-2-oxo-1,3-benzoxazol-3(2H)-yl)-N-methyl-N-phenylacetamide]
GAPDH: Glyceraldehyde-3-phosphate-dehydrogenase
IBA-1: Ionized calcium-binding adaptor molecule 1
i.p.: Intraperitoneal
MRI: Magnetic resonance imaging
NonTg: Non-transgenic
PERMANOVA: Permutational Multivariate Analysis of Variance
PET: Positron emission tomography
Ro5: Ro5-4864
TauTg: Tau transgenic
TBS: Tris buffered saline
TSPO: Translocator protein
TYROBP: Tyrosine motif binding protein
Veh: Vehicle
