# Supplementary figures and images for: Neuroprotective effect of mitochondrial translocator protein ligand in a mouse model of tauopathy
Source: J Neuroinflammation. 2021 Mar 19;18:76. doi: 10.1186/s12974-021-02122-1 (PMC7980620; doi:10.1186/s12974-021-02122-1)

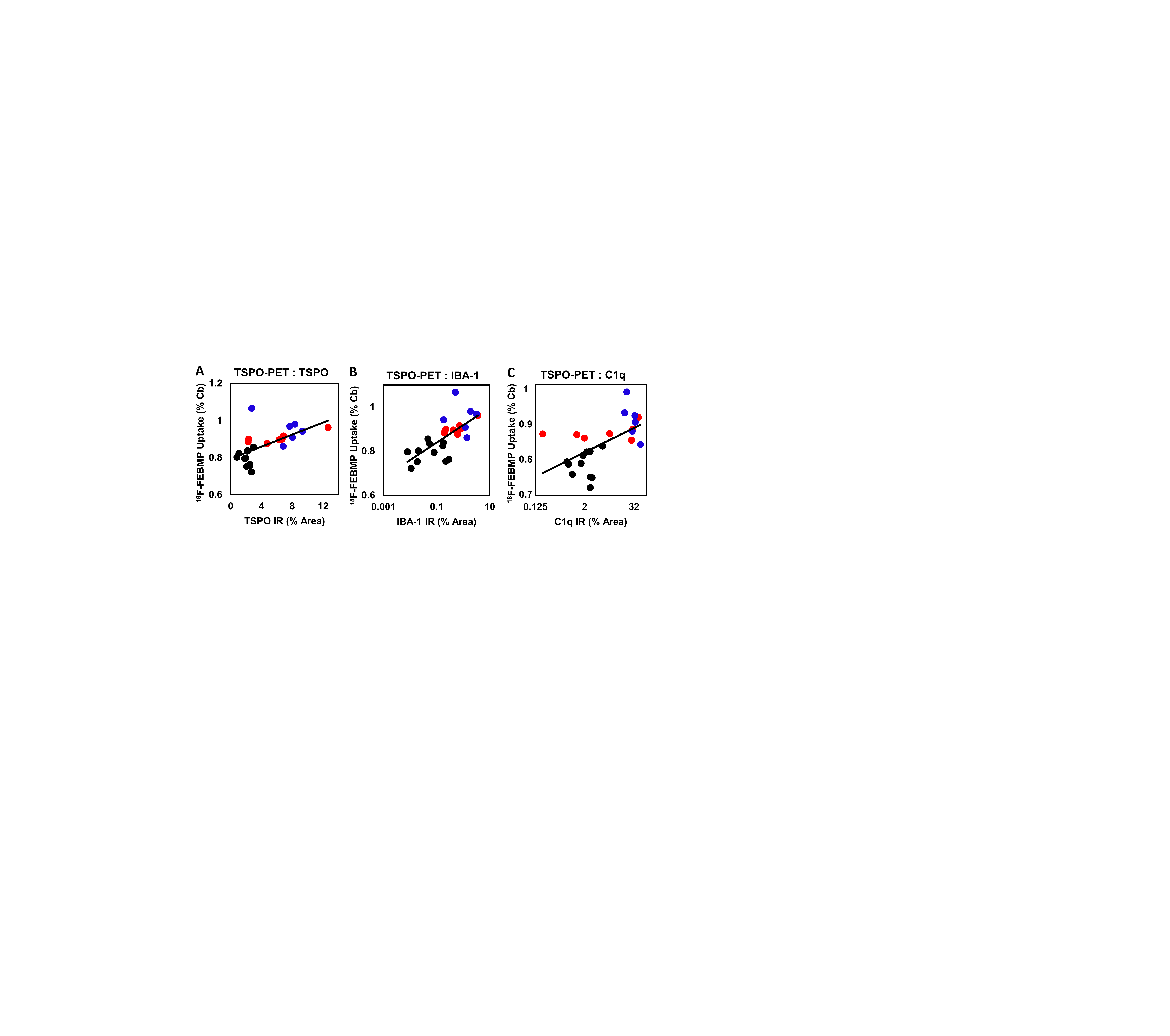

Supplement: Supplementary file 1 — Additional file 1: Supplementary Figure 1. Association between in vivo hippocampal TSPO signals measured using 18F-FEBMP-PET and inflammatory marker immunoreactivity. 18F-FEBMP-PET signals and immunoreactivity (IR) of TSPO (A), IBA-1 (B) and C1q (C). [file 12974_2021_2122_MOESM1_ESM.tif]
